# The Etruscans: Setting New Agendas

**DOI:** 10.1007/s10814-021-09169-x

**Published:** 2021-10-26

**Authors:** Charlotte R. Potts, Christopher J. Smith

**Affiliations:** 1 Ioannou Centre for Classical and Byzantine Studies, University of Oxford, 66 St Giles’, Oxford, OX1 3LU UK; 2 School of Classics, University of St Andrews, Fife, KY16 9AL Scotland, UK

**Keywords:** Etruscan, Etruria, Urbanization, Knowledge exchange, Religion, Literacy, Architecture, Dissemination

## Abstract

The Etruscans, who dominated central Italy for much of the first half of the first millennium BC, are ripe for new analysis: the quantity of data for their culture is now substantial, wide ranging, and qualifies for large-scale comparison. In this paper, we survey how research in the last decade has affected our understanding of settlements, of changing models of the transfer of ideas, and of Etruscan religious behavior, among other topics. We place them into complex spatial, architectural, and economic narratives to show that the interplay between microhistorical case studies and macrohistorical trends has now achieved what ought to be a paradigmatic status. Despite the continuous flow of specialist publications and an industry of exhibitions, however, the Etruscans have not broken through into mainstream archaeological awareness. We argue that this could be achieved if future research becomes more thematic and agenda driven and embraces comparative study.

## Introduction

**Fig. 1:**
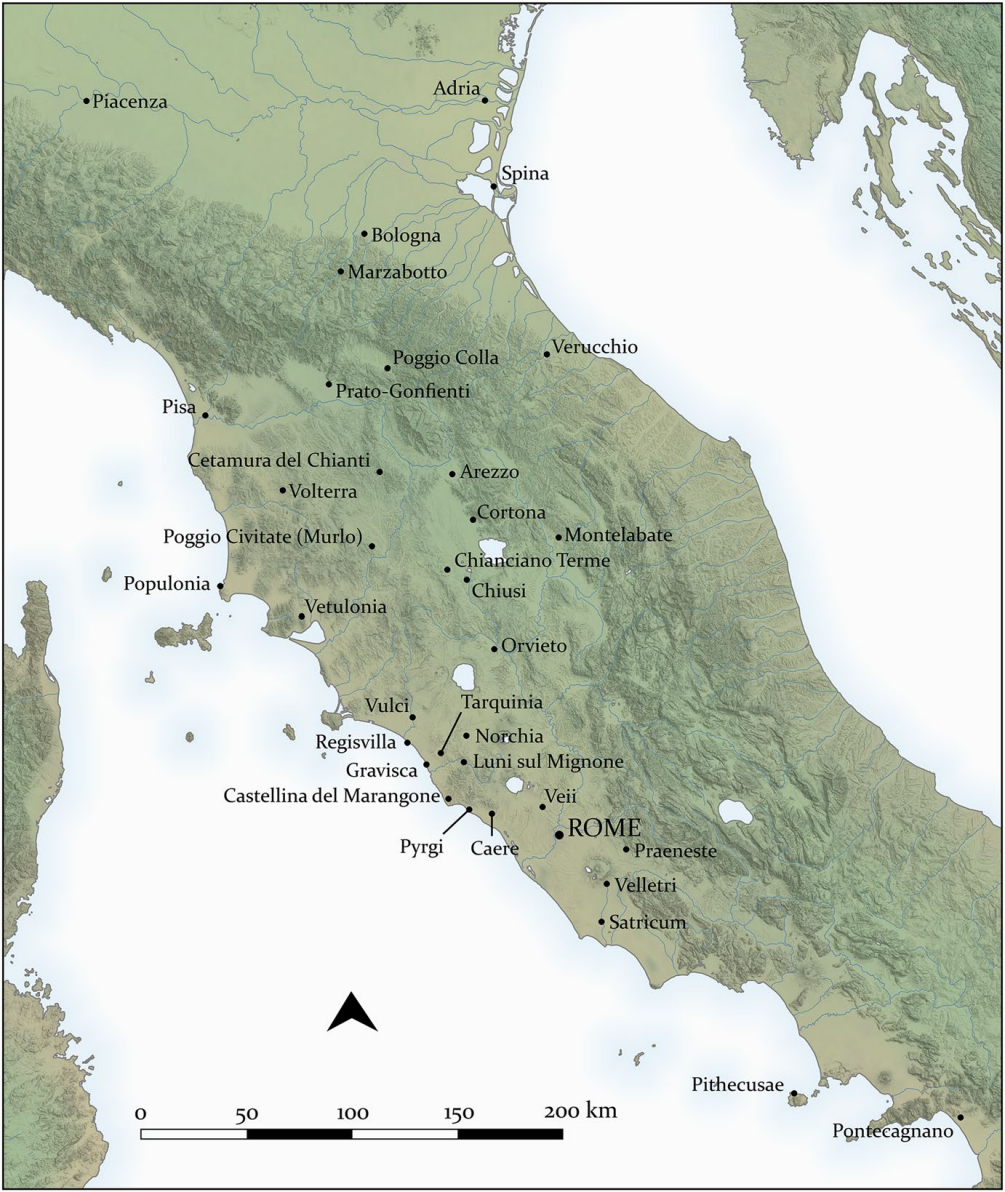
Sites mentioned in the text.

The Etruscans occupied a region of central Italy between the Apennine Mountains and the Tyrrhenian Sea (Fig. 1). Their most characteristic presence was in the area roughly between Rome and Florence, a fertile area also characterized by significant mineral resources. A more or less distinctive linguistic and cultural community can be identified from around the beginning of the first millennium BC, and they expanded significantly into northern Italy and south into Campania. The latter expansion was curtailed in the fifth century BC by Greek settlers in the area, and Roman imperial growth in the third and second centuries BC restricted Etruscan political independence. By the end of the first millennium BC, the Etruscan language was largely extinguished, their literature lost and decreasingly known, and their culture substantially transformed into the general Roman model. The Etruscans, thus, are the most significant example of an Italic grouping that flourished, and indeed for a while eclipsed Rome, before fading from view, until they were recovered through early modern antiquarian interest and more modern archaeology.

This is a good moment for reconsidering the Etruscans. The quantity of data is now substantial across all ranges of material and qualifies for large-scale comparison. This is especially true when we consider the understanding of settlements and settlement dynamics. As we start to think increasingly seriously about how to understand the transfer of ideas, in the context of decolonized curricula and reanimating the agency of all players across globalized landscapes of connectivity, the changing models of “Orientalization” in central Italy offer new models. Interest in ritual and religion in societies with limited literacy, or where such evidence has not survived, is a topic of general interest in archaeology and anthropology. Etruscan religious behavior is of central significance to an understanding of their society, and analysis has reached a high degree of sophistication. We can also insert these narratives into more complex spatial, architectural, and economic analyses and see that the interplay that is now possible between microhistorical case studies and macrohistorical trends has achieved what ought to be a paradigmatic status. However, despite the continuous flow of specialist publications and an industry of exhibitions, the Etruscans have not quite broken through into mainstream archaeological awareness, and that is partly to be laid at the feet of a scholarly tradition that has tended to isolate itself from wider theoretical and comparative trends. Much scholarship remains concerned with typologies, iconographies, and the exposition of detail. This article seeks to continue the work of drawing attention to the Etruscans by adopting a more thematic and agenda-driven organization and, thereby, to show how tackling the largest questions at a local and regional level can illustrate the potential for comparative study.

The Etruscans have been the focus of study for centuries; already in the Renaissance, they were a specific and distinctive part of the revival of interest in the classical past, and they have been continually deployed in complex arguments over autochthony, distinctiveness from Rome, and originality (Riva 2018). Contemporary study of the Etruscans, however, differs significantly from that undertaken in previous centuries. Even the remit of the subject has changed. In the 19th century, in comparison with the difficult site of Rome and the almost unknown world of Latium, material from Etruria was relatively abundant, which meant that Etruscology once held prime significance in the study of early Italy (Della Fina 2011; Haack and Miller 2015). Now the physical and cultural boundaries of the discipline are being increasingly challenged. More and more the Etruscans are seen within wider and nonhierarchical accounts of the peoples of Italy (Bradley and Farney 2017); for example, the journal *Etruscan Studies* has been renamed *Etruscan and Italic Studies.* The extent of new material from Rome has given that city an identity progressively separated from that of its Etruscan neighbors; for instance, the beginnings of “Roman” architecture have now been moved back into the Archaic period, instead of being regarded as purely derivative (Cifani 2008; Hopkins 2016). The range of scientific tools used to study the Etruscans has also grown. The fascinating and unusual Etruscan language has always been of interest, but our tools are arguably better now than ever before (e.g., Wallace 2008, cf. Bellelli and Benelli 2018). Closer work with archaeological sciences such as zooarchaeology, archaeobotany, dendrochronology, and petrography, particularly in the study of periods with little or no textual evidence, is expanding the field in ways comparable to the introduction of landscape surveys in the 1950s. Research on topics such as archaeozoology and palaeobotany (Trentacoste 2016; Trentacoste et al. 2020; Trentacoste and Russ 2021), DNA (Perkins 2017), textiles (see below), and technological capacity (e.g., Amicone et al. 2020; Ceccarelli et al. 2020; Weaver et al. 2013) has likewise pushed the subject beyond its historic focus on typology, individual sites, and individual classes of material.

In the last decade, the relationship between Etruscan studies and other areas of classical archaeology has also seen continued renegotiation. Whereas Greece and Rome, with their rich literary traditions, have offered models in theory with wide applicability, the Etruscans, whose literature has not survived, have tended to be forced into others’ models instead of constituting their own theoretical space. This has not been helped by a tendency on the part of modern scholarship to remain more comfortable with established approaches rather than moving to higher levels of analysis, comparison, and explanation. Uneasy balances between old and new approaches, and between how topics in Etruscology are framed in contrast to those in Greek and Roman studies, have emerged together with new research questions, all further challenging how the field sees itself. Ultimately, we would argue, the most productive future for Etruscan studies may lie in their capacity to enter a more fruitful dialog with Mediterranean and wider archaeology.

We attempt to move the subject forward by identifying key areas in which knowledge is shifting and where the study of Etruscan material offers opportunities to lead and define paradigms rather than follow them. Our review is not and cannot be comprehensive, as the volume of work over recent years is staggering. Nor do we seek to replicate the helpful occasional series of surveys of Etruscan material published in the *Archaeological Reports* (e.g., Gleba 2009; cf. Zaccagnini and Mercuri 2014), the annual *Convegno Faina* at Orvieto, and the chronologically broad-ranging series, *Preistoria e Protostoria in Etruria*. We wish instead to draw attention to some of the most significant areas of development, to current debates, and to illustrate potential points of import, crossover, and benefit for other fields in classical archaeology and beyond.

## What Has Changed?

In this first section, we map out some of the key type sites and how our understanding of cities, minor centers, and necropoleis has changed over the past 10 to 20 years. The intensive exploration of Etruria over centuries means that it is unlikely that there are many large cities yet to be discovered, but recent work has nevertheless significantly expanded the range of evidence. What this section shows is both the phenomenal wealth of material and also the limitations of the very concept of an Etruscan site. At the heart of the notion of the Etruscans as a discrete ethnic group and to some extent an independent area of study is the enigma of huge variation.

### Cities

Cities are a core feature and organizing principle of Etruscan studies (Bruni and Barbieri 2010; Torelli and Moretti Sgubini 2008). The sources suggest that the Etruscans were once organized into a league of 12 cities—as attested by the Roman author Livy (1984 [c. 60 BC–AD 15], 7.21)—but a list of members has never been agreed and they may have fluctuated over time, while contenders cannot always be equated with the physical evidence either. The large site of Gonfienti near Prato, for instance, has not yet been convincingly identified (or properly excavated or published; Poggesi et al. 2010). The league is said to have held meetings at the Fanum Voltumnae, which may now have been discovered near Orvieto (see below). Beyond this, much about urban organization, hierarchies, and coordination remains unresolved, and theoretical approaches to Etruscan urbanism still have room for development (see Stoddart 2020a). We summarize here some of the major advancements.

In southern Etruria, several of the major settlements have been the object of renewed study. Most recently, Stoddart (2020b) has offered an overall account based on what he calls spatial dynamics, on the establishment of power and on its territorial consequences through time. This approach, depending on models such as rank size, seeks to place Etruria firmly in the broader comparative literature and depends on the increasingly sophisticated results of survey (Campana 2018 for the important Emptyscapes project, http://www.emptyscapes.org/).

Our knowledge of Veii in particular has greatly increased (Tabolli 2019). The long-awaited publication of the British School at Rome excavations and survey have given the site important contextual information (Cascino et al. 2012, 2015). The settlement grew rapidly from the early ninth century BC, presumably benefitting from the trading opportunities offered by the Tiber River. The sequence of activities at Piazza d’Armi, part of Veii’s settlement, is critical, as it offers one of our best insights into the long-term commemoration of a burial site predating the classical period. Here a male inhumation during the early ninth century BC seems to have been a catalyst for what would become a lasting focus of veneration. A hut was built at the site before the mid-eighth century; it was then rebuilt in the eighth and seventh centuries with an additional residential structure, before being replaced by buildings with *tufo* foundations and then abandoned in the fifth century. An additional burial in the eighth century BC, as well as evidence of offerings and markers of burial, establish that the area was a cultic center for more than four centuries (Bartoloni and Sarracino 2017).

The Early Iron Age necropoleis at Veii continued to be used in the eighth to seventh centuries, and tumulus tombs began around 650 BC. The site was already important as a manufacturing area, and we are beginning to learn more about the density of artisanal activity (Cascino 2017). We have also gained substantial information about the road network on the site and the organization of the eventual city (Guaitoli 2015); politically, the site is thought to have evolved from a center organized by districts into a unitary urban center by the sixth century BC. The development of Veii’s territory also shows close interconnection between the city, rural areas, and the river. Of particular significance was the discovery in an Orientalizing necropolis at via d’Avack of a ceramic vessel (a *kotyle*) with a representation of horses being transported by ship (Arizza et al. 2013), a rare and intriguing hint at some form of transportation or trade, perhaps even related to the river itself. We now have an impressive catalog of sixth to fourth century BC tombs (Arizza 2020) showing a gradual reduction in funerary expenditure.

The aftermath of Veii’s destructive encounter with Rome in the early fourth century BC is one of the most significant problems in Veientine archaeology. Our sources describe a catastrophic siege, defeat, and destruction in 396 BC; archaeologically the site appears to continue, at a lower level of intensity, picking up again between 350 and 250 BC, and then tailing off. By the late Republic and early empire, the site is predominantly characterized by villas and possibly a healing sanctuary (Fusco 2019; Liverani 1987).

For all that Veii has been one of the most intriguing and consistently studied sites in Etruria, it is not clear that it is typical or that we should even seek a single urban typology. Not only do we see different kinds of evidence from different sites, but each site occupied and exploited a distinct ecological niche. In the case of Veii, for example, it is possible that it developed a significant territory among the Faliscans, which would make it one of the more “land rich” states (Cifani 2013). The variety of what we call “Etruscan” is enormous, as the following brief survey of other key sites illustrates.

Caere (Cerveteri) was the subject of an important exhibition in 2014 (Gaultier et al. 2014) and continues to see intensive archaeological investigation at its port site of Pyrgi. We now have detailed reconsiderations of older excavations coupled with intensified new activity that has revealed additional monumental buildings from the sixth century and a better understanding of the edges of the site and how exactly it connected to Caere (Baglione and Gentili 2013; Baglione and Michetti 2017). The two major temples from the sixth and fifth centuries at the site are well known, and an Archaic monumentalized area has now been unearthed to the north. Particular attention has been given again to the famous bilingual Etruscan and Punic inscription from the late sixth or early fifth centuries BC about the foundation of a sanctuary by Thefarie Velianas (Bellelli and Xella 2016; Michetti and Baglione 2015). It now seems likely that this represents specifically Carthaginian interest in the site at approximately the same date as the Rome-Carthage treaty of 509 BC. Many parts of the town itself remain largely unknown, but we do have a good new summary of the available data, including in the territory (de Grummond and Pieraccini 2016).

At Tarquinia, there have been exciting developments for our understanding of the town with the reinterpretation of old geophysical data, and we will soon have a map at least as good as the ones available for Veii and Vulci (Bagnasco Gianni et al. 2018a, b; Marzullo 2018). The results of the work at the Ara della Regina temple are indicated later in this review, and it is worth noting here that there is now good evidence of long-term commemoration at the site, as at Veii. Tarquinia’s port site at Gravisca has also reopened and offers the opportunity of ongoing comparison with Pyrgi (Di Miceli and Fiorini 2018).

At Vulci, intensive geophysics by the Vulci 3000 Project have recovered details of the internal organization of the settlement (Forte et al. 2020). Further work is ongoing; we have a new historical survey (Bianchi 2017), palaeoenvironmental work, and excavations at the port of Regisvilla (Regoli 2017) as well as aerial photography (Pocobelli 2010–2011). Populonia is another major urban site, and the discovery of an Early Iron Age hut at Poggio del Telegrafo, which was abandoned and destroyed and not reused for a considerable amount of time, has been claimed as an additional example of long-term memory (Bartoloni et al. 2015). Work on the organization of the acropolis at Volterra is also ongoing (Bonamici et al. 2017; cf. Camporeale and Maggiani 2009), as are new excavations.

Two key sites in antiquity were Arretium (Arezzo) and Clusium (Chiusi), but their distinctive pottery and burials are better known than the settlements. Both were important sites of production, and their economic significance is clear from their wealth, which is mentioned in our sources as much coveted by the Romans (Benelli 2009; Cappuccini 2010). Graves from the Hellenistic period have now been extensively studied (for Arezzo, see Vilucchi 2012; for Chiusi’s famous and distinctive Canopic urns, see Daveloose 2017; cf. De Angelis 2016).

The political organization of the Etruscans farther north is less clear. The southern cities at least for some purposes had a federal structure, but we do not know if this was also the case for the northern cities, and there is still work to be done on the hierarchy of settlement and links between the southern group and the north. One of the most successful urban excavations has been in the unusually regular city of Marzabotto, with a new account of the artisanal areas and the central temple complex (Govi 2017c; Sassatelli and Govi 2005). Work in Bologna (Etruscan Felsina) is naturally sporadic but informative (see below). A team from the University of Zurich is conducting promising work at the complex waterside site of Spina, which is often compared with Venice because of its ingenious architecture and was a notable contact point with the Greek world (Berti et al. 1993); there are interesting possibilities for palaeobotanic work in particular (Reusser 2017). The other aspect of the wider Etruscan influence on the peninsula relates to the settlements of Campania. Here, too, we lack a convincing narrative that makes sense of the archaeological material. Connections across central Italy or by sea seem traceable from the early Iron Age and continue strongly in the Orientalizing and Archaic period, but it is less clear, and much disputed, what kind of political domination we should imagine, and the problem is somewhat analogous to the challenge of understanding Etruscan presence in Rome. What does seem clear is that Greek victories over a large Italic army in 524 BC and an Etruscan fleet in 474 BC off Cumae eroded significantly the Etruscan presence in southern Italy (e.g., Camporeale 2004b; Cerchiai 2010; Cerchiai et al. 2018; Gilotta 2009; Osanna and Verger 2018).

Sometimes, the most intriguing are second-order sites, or sites that fail to grow to full urban strength. One of the best known in Etruria is Poggio Civitate (Murlo) (Coppolaro Nowell 2017). A longstanding excavation project has revealed a set of buildings arranged around an open space in the late seventh century BC, which were replaced by a quadrangular building around a courtyard in the early sixth century. The architectural terracottas from the Archaic phase display scenes of banqueting, horse racing, a cart procession (perhaps a marriage ceremony), and possibly an assembly of gods, as well as striking sculptural decoration including standing and seated male and female figures. The site was destroyed and buried in the second half of the sixth century.

Another site that has become highly visible is Poggio Colla, where the discovery of what is billed as one of our longest Etruscan inscriptions has focused attention on a marginal site located on the path through the Apennines and across the Adriatic coast (Warden 2016; Warden and Maggiani 2020). Work at Montelabate, between Perugia and Gubbio, has also revealed a site that operated very much on the edges of larger settlements. The suggestion that its relative poverty came from the demands of tributary activity (Stoddart et al. 2012) is one of our rare insights into the exploitative nature of the Etruscan countryside. Castellina, between Caere and Tarquinia, offers an important insight into a small site that does continue across the Bronze to Iron Age transition (Gran-Aymerich and Domínguez-Arranz 2011).

We continue to rely heavily on rescue or emergency archaeology and specific interventions. Much work was done on the port at Pisa some years ago, and we now have the suggestion of a small sixth century traders’ enclave (Maggiani 2018). New work is picking up evidence from the important site of Verucchio, but we are still far from understanding the settlement (Harari 2017), or indeed how “Etruscan” it was, and this might lead us to some interesting questions around the firmness of these “ethnic” definitions. The examples could be multiplied. We are gradually beginning to understand more about Etruscan housing (see below), although the extent to which it was “Etruscan” is not clear. Fortifications have been another significant topic of interest, across central Italy, although a better balance needs to be struck between models of defensive necessity and urban rhetoric (Fontaine and Helas 2016; Murata 2008). Finally, field survey and detailed topographical work remains productive; as an example, we note the extraordinary synthesis by Pulcinelli (2016), mapping local settlement and territory in southern Etruria from the fourth to third centuries BC, just before the Roman conquest.

This brief survey serves to highlight one of Stoddart’s key points, namely the presence of substantial hierarchies of settlement type, as well as the wide geographical extent of Etruscan presence and the high degree of local specificity. As a study of urbanism at a regional level, therefore, Etruria both lends itself to the notion of urban networks and disrupts the idea of unified systems in favor of a dazzling array of interdependencies, opportunities, and connectivities. If we turn more briefly to another classic characteristic of Etruscan archaeology, the necropolis, we can see similar levels of variation.

### Necropoleis

Etruscan studies have always been driven by the study of burials. At several key sites it was cemeteries that initially captured imaginations and led to the construction of an image of the Etruscans as a whole. The painted tombs of Tarquinia and the evocative tumuli at Caere are recognizable parts of the Etruscan “brand.”

Work on the methodological problems of working from tombs to society has been plentiful (e.g., Cuozzo 2003; Cuozzo and Guidi 2013; Nizzo 2015; these studies draw heavily on Anglophone scholarship). There are currently few large-scale excavations of necropoleis, but we mention here Colombi’s (2018) important work on an Orientalizing necropolis at Vetulonia, Paolucci’s (2015) work at the Tolle necropolis at Chianciano Terme, and Arizza’s (2020) important summary of material in the ager Veientanus. Burial sites continue to be a major focus of research (Arizza 2020; Morpurgo 2018; Perego and Scopacasa 2016; Tuck 2009). Significant new projects are planned in the Hellenistic necropolis at Populonia, at Crocefisso del Tufo at Orvieto (Binaco and Bizzarri 2018), and work is ongoing at the Etrusco-Campanian site of Pontecagnano (Cerchiai et al. 2018). What is absolutely evident from all this material is that while there are similarities and continuities of behavior, each necropolis also relates powerfully to its local community and the conditions of the site. The existence of striking differences between the aboveground tumulus tombs of Caere and the underground chamber tombs of Tarquinia is replicated across the region.

One type of necropolis that has attracted a good deal of attention recently, and that offers yet another typology, is rock cut tombs (Steingräber and Ceci 2014). Ambrosini’s (2016, 2018) new publications about the site of Norchia have updated our knowledge of one of the most dramatic sites in Etruria, and summary publications have shown how widespread the phenomenon is (Gori 2019). Although one can go far with an analysis based on site morphology and geology, the distinctions between cities of the dead increasingly look as if they mirror the distinctions between cities of the living that we touched on above. We turn now to one of the developments that fundamentally shaped the Etruscan world, namely the formation of the communities that made up its cities.

## Urbanism: When, How, and Why?

One of the classic paradigms for the evolution of society is the emergence of urban communities (see Woolf 2020; Yoffee 2005, 2015; Zuiderhoek 2017). Located at different points in time in different societies, urbanism has remained a powerful model for the shift to more complex and stratified societies, though one derived from a specific Greco-Roman narrative. Alongside a teleology of band, tribe, chiefdom, and state, we find a tendency, which goes back at least to Fustel de Coulanges (Yoffee and Terrenato 2015), to reify the ancient city into an aggregation of population and functions that map on to social complexity.

In Etruria, this is classically placed at the point where Late Bronze Age “proto-Villanovan” villages, which increased in number across the period, gave way to large urbanized settlements across plateaus, which are characterized by Villanovan material culture (Bietti Sestieri 2008, 2012; Negroni Catacchio 2010; Pacciarelli 2016, 2017 Stoddart 2016; Vanzetti 2004). However, this broad statement contains within it a number of problematic labels and definitions that reveal how flawed it is as a description of the Etruscan experience.

### The Emergence of the Etruscans

The term Villanovan derives from a single site, near Bologna, whose grave goods set the standard pattern for southern Etruria; “proto-Villanovan” was simply an attempt to stretch a problematic adjective backwards in time. As is repeatedly stated, there was no such thing as a Villanovan people, only Villanovan material culture, and many have argued that the term should fall out of use and instead be replaced with the label “Iron Age.” The choice of term, however, is bound up with the awkward issue of Etruscan ethnic identity.

Theories of autochthony or migration have long been something of a battleground for Etruscan studies (Bellelli 2012). The general trend is now to emphasize cultural change as the product of internal developments rather than external influence. There are no good grounds for seeing a major population shift either between what were termed the proto-Villanovan and Villanovan periods, or during the Iron Age, and it is very difficult to see why we continue to use labels like proto-Villanovan and Villanovan when we are referring to the population of the area that we call Etruria (Negroni Catacchio 2000). The apparent logic is that ethnogenesis was a product of urbanism. Blake (2014), using network analysis, has suggested that the group that is later regarded as ethnic, in some sense, is visible in the Bronze Age, and while the constructed nature of ethnic identity is an important fact to us, the question remains as to what extent and when ethnicity became a characterizing feature of the Italian world. In other words, the connectivity that unites a particular region in terms of geography, geology, settlement, and people may be much older than the creation of a generic name.

At this stage, the question gets caught up in arguments over when we can identify the urban moment, and also of where identity sits, in the federation or at the city level. To what extent did Etruscans think of themselves as Etruscan, or Tarquinian for instance, an issue that likewise has been raised for the Phoenicians, who may have identified with their cities far more or even to the exclusion of any ethnicity (Quinn 2018). The Etruscans to a degree can be incorporated into discussions about the ethnographic inventions of later periods (Bourdin 2012), which reinforces the idea that it is unhelpful to apply an overarching term such as Villanovan to an earlier period; as a result, many accounts of material culture now operate on a settlement basis. This paves the way for a much more particularized account of the local conditions within which, at more or less the same time, a number of communities left smaller settlements that are visible in the Late Bronze Age landscape and reemerged on larger plateaus; the focus accordingly falls on a process rather than a people. But when did that process take place?

### Chronological Horizons

One of the most critical changes in Etruscology in recent times has been the alignment of central Italian dates, long based on material culture and stratigraphy, with dendrochronological dates in central Europe (Bartoloni and Delpino 2005; Bietti Sestieri 2008; Delpino 2003, 2008; Nijboer and van der Plicht 2008; Pacciarelli 1996; van der Plicht et al. 2009). As a result, the Early Iron Age in Etruria now starts earlier than previously thought. A similar uplift in dates seems to be occurring across the Mediterranean. This means that the large plateau sites came into being earlier. The move away from villages in the Final Bronze Age and the shift to larger sites happened rather more quickly than we used to argue, but the subsequent phase of settled occupation was rather longer. It is also clear that this underlines the importance of local developments—endogenous development instead of external influence.

Understanding the transition from the Final Bronze Age to the Early Iron Age is challenging (e.g., Alessandri 2013). We know that wealth creation was already a feature of society in the Final Bronze Age, visible in hoards of metal, and we suspect that some form of social hierarchy existed in certain settlements. A better understanding of this period and its passing is a key objective (e.g., Barbaro 2010; Pacciarelli 2000, 2009). Perhaps the most important type site of this phenomenon is Luni sul Mignone (Barbaro 2010, pp. 169–172). The apparent disappearance of the Late Bronze Age system, with some 90% of settlements being abandoned, is striking.

If small sites coalesced into larger ones, often incorporating existing settlements, then one critical question is whether social hierarchy carried over from one to the other. Terrenato (2019) recently argued that this indeed happened, with important consequences for understanding the nature of these early settlements and the extent to which they attained anything like urbanism.

### Discontinuous Community

The plateau sites were huge compared with what had gone before. The four major sites in southern Etruria were Veii, Tarquinia, Caere, and Vulci, each between 125 and 175 ha in size. It seems likely that they were discontinuously settled, with nuclei of people occupying separate parts of each plateau, perhaps with some level of common identity across the site; further research is needed to establish how widely applicable this scenario may be. The scale and consistency of this revolutionary movement of the population are striking, and Pacciarelli (2017, p. 573) insists that it must have been the product of a “specific political project.” However, it remains extremely difficult to find an appropriate language to describe this enormous reshaping of the landscape.

Different factors may have been influential or incidental. The evidence from Piazza d’Armi at Veii, where ancestors of a descent lineage appear to have been commemorated, could be read as a sign that forebears played an important role. We are wary of using words like worship here, as it would imply a set of religious and other beliefs that we cannot demonstrate, and it may obscure rituals that were more honorific than concerned with cult, but the evidence is intriguing (see below). In the model developed by Terrenato mentioned above, in which each site contained several sub-settlements carried over from preexisting sites, the commemoration of ancestors could suggest the persistence of successful kinship groups. Yet at the same time, there is significant evidence of relatively large-scale efforts to fortify the new sites (Fontaine and Helas 2016), although the early fortifications at Veii on Piazza d’Armi are now thought to be medieval (Bartoloni and Pulcinelli 2016). Defense—real or symbolic—thus, should not be discounted either.

The contradiction between the two scenarios implied by this evidence, between discontinuous settlement and possible extended family groups as an organizing principle, on the one hand, and communal action in relation to fortification, on the other, is difficult to resolve. Evidence for early central spaces, like those built in Rome between the eighth and sixth centuries BC, would be helpful in assessing level of shared identity, but they are currently lacking with the exception of cult sites like that on the Civita plateau at Tarquinia, which could well have served multiple functions. At present, the models for understanding Etruscan urbanization thus depend heavily on the idea of longstanding elite families and limited evidence for community action.

### The Role of the Ancestor and the Nature of Economic Growth

As time passed, Etruscan burials changed with the introduction of large-scale interments and increasingly elaborate displays of wealth. Again, this raises important questions about the relationship between funerary evidence and society, and between real and fictive kinship. It is commonly stated that Etruscan society was gentilicial between the ninth and seventh centuries BC, but this description needs explanation. As a term, it derives from the Latin (not Etruscan) social formation of the *gens*, but it has developed its own history as a term attempting to convey the importance of large social groups. Recent research has suggested that Levi-Strauss’ house society might be a useful parallel (Naglak and Terrenato 2019, 2020). In Etruria, we remain short of evidence that would strongly substantiate the application of the term for any period before the construction of tumulus tombs in the late eighth or early seventh century (discussed below), which takes us to a different world.

It is precisely because of the length of the long Early Iron Age that this becomes more significant. The models we are choosing between offer different trajectories. One is long-term stability across a period of relatively discontinuous settlements and changeable burial practices. The other is a more fluid and unstable situation in which the deposition of wealth in tombs perhaps represents a combination of display looking back across an individual’s life and restraint looking forward by placing items out of reach. The increasing use of status symbols, for instance related to warfare or banqueting, does indicate the importance of display, and it is clear that capital accumulation, or the creation of value, are present across the period (e.g., Iaia 2016; Riva 2010). The real challenge is the extent to which that value was transmitted across generations. As we suggest below, it is important that we build into this narrative some appreciation of the evolution of architecture and building techniques and their role in society, too.

One hypothetical solution might be to see the small sub-settlements on the plateaus as mechanisms for economic accumulation and the development of necropoleis as a way of mirroring that process, which then became far more elaborate in the seventh and sixth centuries BC, although family units may if anything be smaller. The advantage of recognizing some form of accumulation in this process is that it makes sense of the global stability of the plateau sites, whatever minor changes we may see, and thereby lays the ground for the major shift that occurred around 700 BC.

However, this does return us to a key question about urbanism. Were the main plateau sites actually urban, and at what point? What was their urbanism for, or what did it help the inhabitants achieve? And are the terms “urban” and “urbanism” useful at all in studying forms of social and physical organization at this point? Stoddart argues that we see a series of tipping points marking progressive urban development but that the difference between the late Bronze Age and Early Iron Age was the first and crucial (Redhouse and Stoddart 2011; Stoddart 2020a; see also Di Gennaro and Stoddart 1982; Di Giuseppe et al. 2020). The key process that drove this change was nucleation, and he suggests that it may have resulted from a corporate decision by a band of descent groups to coalesce (the term in the Greek context is “synoecize”) on a defensible site; we might contemplate that defenses and position may have had their own symbolic value. The examples of Tarquinia and Veii can both be used to illustrate this hypothesis. Whether the move was a response to some external factor (such as a change in climate or political or military disturbance) or the internal limitations of the Final Bronze Age system remains to be discovered.

These large settlements, and a gradual proliferation of others over the ninth and eighth centuries BC, began to refill the landscape of Etruria. We should not forget that northern Etruria had a slightly different trajectory, with slightly more and slightly smaller sites (Stoddart 2020a). To a large extent the “Villanovan revolution” in the location and organization of the population created the basis for several centuries of Etruscan settlement. We next consider how external goods and influences coming into this system were deployed to stabilize, and perhaps disrupt, the long Iron Age.

## Orientalization or Knowledge Transfer: An Alternative to Periodization?

By the eighth century, Etruscan settlements had established territories, internal articulations of space, and social differentiation with indications of roles, wealth, and status visible in grave goods. For the most part, weapons and armor for men, and equipment for textile working and feasting for women, are typical, and the increasing quantity of these items along with jewelry and personal accoutrements in burials suggest that society was developing an internal economy (Riva 2010).

Into this socially differentiated and politically evolving world came the transformative impact of an increase in goods and ideas from the eastern Mediterranean. This process and period are still usually described as Orientalizing, largely by convention, and have been intensively studied for the Etruscan world and, indeed, the Greek world too. However, it is also a phenomenon that needs to be broken down and rethought.

First, we should acknowledge that the terms “Orientalizing” and “Orientalization” are now controversial. If we choose to use them, then we should do so as a reflexive process of internal positioning and, perhaps, see them operating at a more granular and individual level than on that of a whole culture (Riva and Vella 2006). Originally, the notion was coined as part of an art historical recognition of the similarity between items found in rich Etruscan graves and artifacts produced in the Near and Middle East, especially in the Levant and Assyria. As such it played into notions of the exotic richness of an undifferentiated “east,” and was principally used to describe funeral display in the late eighth, and especially seventh, centuries. Over time, it broadened into a much wider description of the interactions between east and west (themselves intellectual abstractions often centered on Greece), including influences on language, costume, behavior, and ideas, one of the most significant of which was the spread of the alphabet and writing habits. The dangers of the conventional terms are clear. There was no single Orient but instead a series of interlocking and connected exchange networks along which objects were passed, losing and acquiring meaning over time and space (see Gansell and Shafer 2020). The notion of agency wrapped up in the termination- ization is also problematic: who was being changed and by whom? Seen in a longer chronological context, it is increasingly clear that local elites, and communities more generally, responded to external influence by absorbing and reinterpreting ideas, and allowing them to reproduce or transform local structures. An alternative way of looking at this process is, thus, to see it as a revolution in knowledge and technology.

We might also think about the changes in Etruscan culture as an absorption of grammars of alterity, rather than Orientalization or Orientalizing; that is to say, an increased capacity to incorporate different ways of being and thinking, an enhanced experience of learning from others and applying that learning to one’s own society. We find, for instance, more pottery originating in Greece in cities connected to the coast than inland cities, which tended to have imitations instead (although there also was imitation pottery in coastal cities). This may reflect differential access but also is to be understood in terms of choice and different mechanisms of internalizing external knowledge. At every stage, and in every example, we have to give space for agency, volition, and reinterpretation. “Orientalizing” goods are not just found in extravagantly rich, so-called “princely,” tombs, and although there are generic similarities between these tombs, there are also clear efforts to self-differentiate with exceptional goods like ostrich eggs or glass (Bartoloni and Morigi Govi 2000; Bruschetti 2013; Fulminante 2003; Rizzo 2016). Some tombs have more emphasis on objects such as chariots (Emiliozzi and Sannibale 2018), and there are complex relationships between imagery adopted from outside and integrated in local manufacture and imported or wholly local objects (e.g., Biella et al. 2013; Biella and Giovanelli 2015). Taken together, we can see a range of options for performative and scenographic rituals in contexts from the urban setting to the tomb.

It is also noteworthy that Etruria has particular value for interrogating and undermining the notion of Orientalizing because many of the interactions and transfers of knowledge between the differentiated east and Etruscan cities and individuals were subsequently replicated, albeit in different contexts and conditions, during the sixth century BC in relations between Etruria and southern Gaul. This means that Etruria became, in the old terminology, “the east” for the Gauls, but it transmitted both Greek and Etruscan ideas and objects that were internalized locally. Nor was this the first time that the Celtic world met Etruria, or the only direction in which ideas flowed. Just as the Orientalizing period for central Italy is better understood as a phase within long-term complex multidirectional interactions (Iacono 2019), so we increasingly need to see the relationship between central Europe and Italy as including but not confined to the Etruscans and Greeks (e.g., Sacchetti 2016). As a consequence, we can clearly see multiple instances of knowledge exchange, rather than a single unitary process, and we can see visible outcomes at social, political, cultural, and economic levels (Bagnasco Gianni et al. 2017; Bats 2014; Gailledrat 2015; Gailledrat et al. 2016; Joncheray 2017; Riva 2017).

One example, which predates the period we call Orientalizing and, thus, strengthens the argument to consider changes more broadly, is the development of iron technology and the consequent interface between bronze and iron [iron technology does not *replace* bronze technology, but it accelerates a conversation across diverse materials (cf. Erb-Satullo 2019 for the Levantine Near East)]. The development of iron technology in Etruria may have been assisted and accelerated by the large quantities of iron ore near Populonia, which later became a key export and appeared in the joint Phoenician and Greek settlement of Pithecusae. The consequences of the shift to iron for some objects while bronze was retained for others were both social and technological: a significantly enhanced vocabulary of display was one outcome; another was the potential for a range of functional and reusable tools, as was a more prosaic need for greater quantities of fuel for smelting (Biella et al. 2017; Corretti 2009, 2017; Iaia 2006; Zifferero 2017). It is important to keep in mind that bronze technology and design continued to be extremely strong and innovative during this period, and it is clear that central Europe was one source of inspiration. We see both in the development of sheet bronze (Iaia 2005) and specific objects and iconographies. We should not simply add another process to that identified for eastern influence though; it is precisely the complex mix of factors that is critical and potentially consequential.

Another example is textile manufacture. Eastern influence changed mechanical production and the method of weaving itself and introduced new color dyes, such as purple derived from the murex snail, which were imported and used as status markers (Gleba 2016). To the degree that textile manufacture was a female occupation (and there are some caveats that one might make here, for example, as production scaled up, but it seems to retain some truth), we have another indication of how ideas were introduced across society. Notably, some textile tools were inscribed with letters and signs, which offers interesting possibilities for the possibility of female functional literacy. In the same vein, several “princely” tombs clearly belonged to “princesses,” and chariots have been found in both male and female burials (Emiliozzi 1997).

The relationship between iconography and the flow of ideas is a critical topic, and our understanding of it is clearly hampered by the loss of Etruscan literary evidence (see below). However, it seems clear enough that at least some elements of the mythological world of the Greeks, in particular, traveled into the Etruscans’ own cosmology and influenced their religion; Rochberg’s study (2004) of Mesopotamian divination shows some striking parallels with later Etruscan practice (discussed below). It has been suggested that the Etruscans taste in Greek vase decoration was specific and mythology was not a simple transfer (Bundrick 2019; De Angelis 2016). Yet adaptation is as important as adoption, and it is critical to understand both the internalization and the transformation of ideas in the Etruscan context. The notion of the leader in war and at the hunt may have influenced Etruscan practice, just as we know that the Etruscans adapted feasting and banqueting practices from elsewhere, including the use of wine, accompanying sports, related furniture, and possibly even eastern-style tents, all of which can be located in art, especially in the painted tombs in Tarquinia, or traced through burial goods. All this provided the backdrop to more distinctively local developments. Etruscan boxing was distinctive, and in the fifth and fourth centuries BC the presence of freeborn Etruscan women at banquets was treated by Greeks as scandalous (e.g., Thuillier 1985, cf. Thuillier 2013; see also Lulof and van Kampen 2011; Pitzalis 2011). The capacity of the elite in central Italy to mingle and intermarry further suggests that physical and social agility accompanied intellectual adaptability.

Another area, much studied recently, is that of writing and literacy (Bellelli and Benelli 2018; Bruschetti et al. 2015; Tuck and Wallace 2018; Whitehouse 2020). The Etruscans took over a written alphabet that originated in the near East and then spread across the Mediterranean but put it to their own use. We are beginning to understand not only the shifts in how Etruscan writing was used (some of the greatest variety was early on) but also to think about who used it (scribes seem to have been part of Etruscan society). The ability to read and write are sufficiently visible in high status burials and other contexts to imply that they were an important part of the social persona. Where we can follow this down the social order, we can begin to chip away at the notion that Etruscan society was frozen in an elite-centered model.

From generalized skills to specialized knowledge communities, the impact of broader horizons and the openness to grammars of alterity are also important ways of understanding the political changes that characterized the first half of the first millennium BC; they may be more helpful than traditional narratives of city-state formation. We can even examine that concept. As we saw in the earlier description of the growth and development of Veii, the traditional narrative suggests that Iron Age sites entered the Orientalizing period as separate districts but by the end of it were unified urban entities. It might, therefore, be argued that state formation was a product of Orientalization. Our narrative would reframe this: communities were constantly rethinking the technologies and knowledge required to succeed politically, and these were undoubtedly informed by external models but also were naturalized.

Moreover, the outside was not entirely trustworthy. One of the best attested facts of Etruscan history in the sixth century BC is the participation of the fleet of Caere in a naval battle off the Corsican town of Alalia between 540 BC and 535 BC, as reported by Herodotus (1990 [c. 484–425 BC], 1.166). The participants were Phocaean Greeks who had founded a colony at Marseilles and were expanding, fighting against an alliance of Carthaginians and Etruscans. The Phocaeans were defeated, but the Caeretans murdered their prisoners, and after divine retribution they had to make a dedication at Delphi. So Caere was an ally of the Carthaginians against the Greeks, then offered reparations to the Greeks; the presence of a Punic inscription referring to Thefarie Velianas sharing dedications at Pyrgi, probably with the Carthaginians, suggests a reversion to the earlier association. It is not unreasonable to try to trace a pattern of shifting alliances and affiliations between the Carthaginian and the Greek world in the sixth and fifth centuries, as the politics of the western Mediterranean evolved (Bellelli and Xella 2016; Bernardini 2001).

Eventually, this fluidity met the barrier of Rome. Our sources are clear that Rome benefitted from Etruscan contact: architectural innovation, religious practices, customs including the triumph and the toga, and even some of Rome’s kings are thought to have come from Etruria (Bradley 2020; Della Fina 2009, 2010). This is a story of knowledge transfer, but it is the prelude to Roman conquest. Rome’s military dominance in the peninsula is indisputable. But what was the impact?

For some decades now, the notion of “Romanization” has been defended and disputed even more fiercely than Orientalization, and for some of the same reasons (e.g., Aberson et al. 2016; Aberson et al. 2020; Keay and Terrenato 2001; Torelli et al. 1995). Where is agency? Was it a uniform process or distinct in different levels of society? The balance between the loss of political freedom along with much else, and the space for individual betterment, is difficult for modern scholarship to get right, but our model might offer some help even here. There is no doubt that there was some ground for exchange, for the development of local power structures and practices within the overarching fact of Roman control. Etruscans also rose to positions of significance, none more so than the emperor Augustus’s friend, Maecenas, whose Etruscan ancestry was famous, and there was a lingering cultural cachet in Etruscan studies, as shown by specialists from the senator Nigidius Figulus to the emperor Claudius (Chillet 2016). We should ask how people at the time conceived of what was happening as much as what word we should use to describe it today.

In the second and first centuries BC, we can see the elite playing games of cultural bricolage, setting Etruscan and Roman ideas alongside each other and seeking interesting juxtapositions. An interesting example is the way that the Spurinna family at Tarquinia interwove their family’s history into that of the town’s (Torelli 2019). Roman power seems to have enabled the Etruscan elite to acquire a certain stability of local authority, even as they endeavored to make displays of independence. Here, too, we see grammars of alterity, in a way, but perhaps with diminishing scope. For whatever reasons (political dependence, military impotence, stiffening social hierarchies, or tacit discouragement), the capacity for the Etruscans to be visibly different from the Romans lessened. Their language was lost, their villas came to look like other houses, and Etruscan names fell out of use. There was a reduction in the capacity to absorb and transform knowledge; there were fewer chances to deploy a grammar of alterity. This diminution of symbolic language may have been counterbalanced in various ways, some of which are beyond detection by archaeology.

We have taken the story to the Roman period precisely because we want to show that the various processes that had preceded it—Orientalization, Hellenization, even the early phases of Romanization—all had permitted the existence and flourishing of an Etruscan identity, which can be located in performance and in knowledge communities. One of the most characteristic of these is religion.

## Etruscan Religion: A Constant or a Variable System?

Religion is one of the most celebrated aspects of Etruscan culture, not least because of the way it was invoked by ancient authors and the wealth of surviving evidence for its practice. It is challenging, however, to trace beliefs and actions over a millennium of change, and there has also been a historical preference for analyses of one type of evidence or ritual, at a single site in a specific period, rather than syntheses exploring larger theological questions. Here, we take a broader view and argue that our understanding of Etruscan religion could be refined by engaging with debates in related fields, and that our data in turn offer models that could usefully be applied in the study of Greek and Roman religion, of the archaeology of religion, and in the anthropology, sociology, and economy of religious practice. Comparison is key to moving the subject forward.

Etruscan religious studies tend to fall into two related categories that organize our discussion. The first concentrates on the remnants of cult activity preserved in the archaeological record; the second focuses more on the beliefs that drove those expressions and their role in society. The concrete and the abstract are mutually reinforcing, yet different evidence has tended to be employed in each area of study, with important implications for reconstructing the subject as a whole.

### The Archaeology of Religious Practice

Material remains of religious action abound in Etruria despite the fact that much of Etruscan religious experience undoubtedly left no trace. Early examples take the form of offerings in caves, underwater, and in the ground; over time, they are joined by evidence from tombs, of cult buildings, votives, altars, sacrificial remains, and inscriptions, as well as representations of deities and rituals in art. Votive deposits and sanctuaries concentrate data, and decades of excavations at prominent sites have only uncovered a small portion of the available material. The volume of evidence justifies interim and summary publications together with edited volumes (e.g., van der Meer 2010) to highlight points of intersection.

Sanctuaries connected with the cosmopolitan cities of southern, coastal Etruria are sites of ongoing excavation and study. The relocation of communities in the early phases of Etruscan urbanism precluded the regular formation of cult sites with deep histories like those in Greece, but there is evidence for some continuity of cult or memory within the first millennium BC at select sites. For example, comprehensive publication of the Archaic phase of the Ara della Regina sanctuary at Tarquinia suggests commemoration over centuries: when a new terrace was built in front of the temple in the fourth century BC, care was taken to incorporate features marking the location of an Archaic chest and precinct wall below (Bonghi Jovino and Bagnasco Gianni 2012). The excavators propose that the chest may have been thought of as a cenotaph for Tarchon, the legendary founder of the city. Such actions seem to represent an awareness of sanctity that lasted through multiple episodes of remodeling.

Veii, too, has traces of potential legendary founders and celebrated forebears. Terracotta statues from the fifth century BC in the sanctuaries of Campetti North and Campetti South that seem to represent Aeneas and Anchises suggest worship of the Trojan hero at Veii, perhaps as part of a connection with Lavinium that can also be seen in inscriptions at both sites naming the same person (Colonna 2009; Torelli 2011). Farther southeast over the plateau, atypical Iron Age burials at Piazza d’Armi became a site of long-lasting ritual activity (Bartoloni and Sarracino 2017). Such finds are furthering discussions about the veneration of individuals and the creation of power, again highlighting the difficulty of distinguishing between funerary, religious, and civic data in early Etruscan history.

Finds at inland and northern sanctuaries are adding depth to accounts of religious activity that go beyond elite families and gifts that could illustrate histories of art. Recent discoveries at the Campo della Fiera sanctuary near Orvieto, the likely site of the Etruscan federal sanctuary known as the Fanum Voltumnae, include a c. 84-cm-high molded trachyte block that served as a statue base and was inscribed to record a donation by a woman—perhaps a freedwoman named Kanuta—from the Laracena family in the late sixth century BC (Stopponi 2011). Farther north, finds at Poggio Colla include a *bucchero* sherd from the second half of the seventh or the early sixth century BC stamped with what may be the earliest scene of childbirth in European art (Perkins 2012) and a sixth century sandstone stele bearing one of the longest Etruscan sacred texts yet known (Warden 2016). The artisans’ sanctuary of Cetamura del Chianti seems to have been continuously occupied between the seventh century BC and late antiquity and shows that the conflict between Rome and Carthage, as well as the “Hellenizing” trends that elsewhere marked the last three centuries BC, largely bypassed the site (de Grummond 2017, 2020). Projects like these are providing data about the lives of women and craftworkers, about the ongoing use of sanctuaries in times of Roman expansion, and, thanks to an upswing in zooarchaeological and archaeobotanical studies, about the landscape, flora, and fauna (e.g., de Grummond 2020). The result is a fuller picture of religious practices and their settings.

Epigraphy emerges from these studies as a subset of archaeological data with important information about the religious actions of individuals and the systems in which they were conducted. Words and phrases were written on votive objects from roughly the second quarter of the first millennium BC onward, and while Etruscan words often defy precise translation, it is still possible to identify common terms that seem to be relatively secure references to acts of giving, dedication, the gods, and sanctity (Maras 2009). So far, the study of such inscriptions has usually prioritized them as texts rather than artifacts—Bagnasco Gianni (1996) is an exception,—although study of the latter would repay attention, exploring aspects like the location of inscriptions on objects and how inscriptions impinge (or not) on other decoration. Connections between writing and religion—for instance through record keeping as a source of authority, or as would be found if sanctuaries like Portonaccio had their own scribal schools (e.g., Colonna 2019)—would entail us constructing better bridges between philology, archaeology, and religious studies.

### Beliefs, Concepts, and Institutions

Moving from physical evidence of religious activities to the beliefs that prompted them, and to understanding the effects of both on society, is necessarily an exercise in hermeneutics. Again, analysis is not unidirectional: objects and actions have the power to shape perception, and we should be wary of viewing material evidence as a substitute for texts if the appropriate technique is identified. The chronology of the evidence at our disposal is also critical. While votive deposits, architecture, burials, and so forth reflect religious practices in the first and second quarters of the first millennium BC, written evidence often dates much later. Those interested in beliefs and the functions of religion prior to the third century BC must grapple with the reality that much of the evidence is archaeological, and its interpretation demands not just recovery and description but appropriate methodologies to recover use and meaning. Of course, believing that it is possible to go beyond description is itself a methodological standpoint, but it is the process of moving from the tangible to the intangible, largely in the absence of literature, which makes Etruscan religion such a rich laboratory for religious historians.

The challenges involved in passing between practice and belief, and the inherent limitations of both the data and possible findings, may partly explain why scholarship has drawn so heavily on written material despite the fact that most comes from the Roman periods of Etruscan history and via outsiders. Mentions of ritual books, fragmentary texts referring to prophecies, and calendrical notes on repurposed linen all date from the third century BC at the very earliest, and require source criticism that recognizes their (generally late Republican) context (Smith 2019b). Livy’s quote about the peerless religiosity of the Etruscans (1984 [c. 60 BC–AD 15], 5.1.6) also repays consideration from this perspective. Read in context, it can be understood as an example of Livy engaging with Caesar’s descriptions of the Gauls and, thus, participating in a type of ethnographic comparison common in Roman history (Maras 2017). Acknowledging that much of this type of material is late, etic, and at least partially the product of historiographical, political, and literary imperatives means one can be skeptical about the extent to which it usefully records information about religion in the first, second, and third quarters of the first millennium BC, while allowing for elements of codification and refinement.

Combining archaeological and literary evidence to reconstruct Etruscan religion as a set of enacted beliefs will only ever produce a partial picture. The image we have at present is more complicated than the older idea that Etruscan religion was a monolithic system controlled by aristocrats, books, and fixed perceptions of the world. Instead, room is being made for temporal change and flexibility, while the balance between a core set of Etruscan religious beliefs and practices on the one hand and heterogeneity on the other is being reevaluated. The result is a contingent group of ideas about the gods and the world, held by different Etruscan communities and factions, in certain places and times, which changed over the course of the first millennium BC. At this point one can also question whether the notion of religion as a system may impose modern ideas on the ancient evidence. In light of work by scholars of religion arguing that interactions with gods did not necessarily constitute a sharply delineated sphere of activity or thought in antiquity (in contrast to the sacred-secular divide introduced later), both “religion” and how it did or did not form a “system” can be interrogated. This is another area in which theoretical models of religion could valuably be brought to bear on the Etruscan evidence. What we are trying to conceptualize with these terms can be broadly broken down into beliefs about the nature of the gods, about how to interact with the divine, and about the organization of space.

Etruscan religion always appears to have been polytheistic. By the Archaic period one can speak of a pantheon and anthropomorphic gods, although this is often reliant on evidence seen through a Hellenizing filter given extensive Etruscan and Greek contacts at the time; some evidence indicates that Etruscan gods had more fluidity in gender, age, and number than their Greco-Roman counterparts. Inscribed votive objects from the Archaic period similarly imply that the gods were then thought of as privileged mortals, given that the formula of inscriptions used to record gift exchanges between elites in the Orientalizing period appears to have evolved into the formula for votive dedications in the Archaic period (Maras 2009). Another change in the formula used to make gifts to the gods during the fifth century BC may signal an additional change in the perception of the gods or of the act itself.

Communicating with the gods seems to have been a two-way process. The longevity and popularity of votive offerings in Etruria encapsulate a belief that supernatural entities were materialistic and were left objects presumably in entreaty, gratitude, pacification, or simple acknowledgment; Roman notions of *do ut des*, or vows made and discharged, cannot be assumed. Dedications to different gods in the same sanctuary open the possibility that a cult place facilitated communication with multiple entities. Divination also appears to have played an important role in Etruscan religion. Discovering the will of the gods by haruspicy (the examination of entrails), the casting of *sortes* (lots), and using thunder, lightning, and mirrors, among other tools, appear in the sources, but the dating of such practices is not straightforward. For instance, the Etruscan brontoscopic calendar, a way to divine the meaning of thunder on any given day, may have been composed between the eighth and seventh centuries BC (Turfa 2012) or between the fourth and second centuries BC (Ampolo 1990–1991). Artistic representations help establish the existence and conduct of different methods at the time of execution, but it is possible that underlying concepts endured while methods changed and vice versa.

Another element that has been thought to characterize Etruscan religion, now under review, is the ritual conception of space. Ancient authors recount that the Etruscans divided the sky or heavens into 16 different regions each occupied by a god, ostensibly to facilitate communication between mortals and deities through portents or worship. The organization of this heavenly scheme, or *templum*, has previously been reconstructed by Etruscologists on the basis of markings on a bronze model of a liver from Piacenza, dated to c.100 BC, and on descriptions by Cicero, Pliny, Servius, and Martianus Capella dating between the first century BC and the fifth century AD. This combination has produced three conflicting reconstructions that principally differ on the location of north. One way to reconcile the differences may be to differentiate between a fixed terrestrial scheme and a movable celestial one that rotates by two sectors per season (Stevens 2009). If correct, this proposal opens the possibility that sanctuaries could be oriented to the seat of a set of gods rather than just one, again questioning the notion of exclusive cult. An alternative solution may lie in differentiating between daily and annual movements of the sun (Pernigotti 2019).

At the same time, the physical organization of some sanctuaries and settlements is being explored as a product not of organic development or applied geometry but according to astronomical alignments. It has been proposed that the orientation of temples at Tarquinia and farther afield may have been determined by the location of certain stars and constellations, in ways that echo the astronomical and solar events thought to have shaped the layout of the new city of Marzabotto in the late sixth/early fifth centuries BC (Bagnasco Gianni 2013; Pernigotti 2019). More work is needed to investigate how such factors might intersect with other concerns such as visibility, topography, and spatial mechanics (Meyers 2013), as well as the *templum* (Bagnasco Gianni in press), and whether beliefs about the alignment of earthly and heavenly space were a lasting feature of Etruscan religion or one adopted more sporadically (Govi et al. 2020 on Marzabotto; cf. Moser and Hay 2013).

Spanning all three topics is the matter of the extent to which Etruscan religion adopted, adapted, and incorporated ideas and practices from other cultures. As we discussed earlier, interaction with other societies was always a feature of Etruscan life, and it is not surprising that points of contact can be identified in respect to religion as much as writing, architecture, and art. The brontoscopic calendar may follow the divination literature of the Levant or Assyria; haruspicy may borrow elements from earlier Assyro-Babylonian extispicy; some members of the Etruscan pantheon, as well as their anthropomorphism, likely reflect contact with Greece; and the rising use of anatomical votives from the end of the fourth century onward may be connected with similar phenomena in Greece and Rome. One can speculate that correspondences like these were a convenient way to foster connections with outsiders, akin to a costuming of indigenous Etruscan beliefs, or alternatively as a signal that Etruscan religion was a (conscious or unconscious) medley of ideas from elsewhere woven into the conceptualization of local spaces and stories. Debates about adoption and adaptation, motive and use are again relevant.

To date, comparative approaches have largely been eschewed in the study of Etruscan religion despite the ongoing debates in Etruscan studies over degrees of external influence. In conferences and books on classical religion—e.g., on sanctuaries, sacrifice, and divination—Etruscan data have often been neglected and, thus, excluded from analyses of classical experience. Exceptions typically occur in studies of Mediterranean practice (e.g., Kistler et al. 2015), but even here much remains to be done. There is particular potential for critical reexamination of the tradition around Roman borrowing from Etruscan religion. In showing how it is possible to study religion in the relative absence of texts, Etruscology should be prominent in discussions about the archaeology of religion and the value of archaeological and historical data for generating and testing theoretical models of religion. In many ways it has the potential to further push the study of classical religion away from a scholarly dependence on exegesis and to ground it in lived, quotidian, and communal experience.

Scholarship analyzing Etruscan religion in the lives of its adherents includes exploration of religion’s social role. The economics of religion have received recent attention, with the consequence that sanctuaries are now seen as economic engines, particularly after increased investment during the Archaic period. In these studies, buildings and statues no longer overshadow the kilns, furnaces, molds, items used in textile work, and weights and measures found at cult sites. The ways in which religion could transform value have also been considered. Rituals could confer and remove symbolic value, while ritualized actions appear to have involved a host of visible and invisible commodities that were constantly exchanged and renegotiated (Moser and Smith 2019). A third approach has examined the relationship between religion and sociopolitical institutions from the Iron Age to the Archaic period in a range of case studies (Govi 2017b). Here the organization of cities, both in terms of governance and layout, appears interwoven with religious concerns from an early date in ways that are evident in the manipulation of space and community. These types of studies of religion are, thus, contributing to our understanding of Etruscan society in its fullest sense.

Together these points show that knowledge of Etruscan religion is far from settled. The next step is to augment reappraisals by engaging with debates in related fields—on topics like representation, embodied belief (cf. Warden 2012), the power of ancestors, domestic and familial worship, and the utility of models such as polis religion and lived ancient religion—where the benefits can go both ways. It is also important to situate religion spatially in the built environment.

## Etruscan Architecture: How Did the Built Environment Shape Society?

For decades, it has been clear that knowledge of the Etruscan built environment no longer relies solely on data from cemeteries. Excavations in cities, sanctuaries, ports, the countryside, and necropoleis have identified houses, temples, farmsteads, tombs, and infrastructure, and improved knowledge of elements that span different types of buildings such as techniques and materials. The result is an area of study that is becoming increasingly sophisticated and starting to be recognized outside classical archaeology.

There is still potential to go beyond technical and esthetic analyses, however, and bring architecture to bear more on the study of Etruscan society. Just as urbanism is a proxy for studying social and political change, individual buildings and the relationships between them offer a way to study knowledge (transfer) and worldviews, as the designs of buildings express and reinforce the values of their designers and users. The built environment also creates the spaces of daily life and thereby molds behavior. It has a role in generating place and identity and creates narratives and memories. Architecture, thus, goes farther than many other forms of art in actively shaping culture. As an instantiation it has documentary value, and as an agent it has historical force.

Bringing these different strands together to increase understanding of Etruscan architecture as a practice and a source requires a framework tailored to local circumstances. As usual, geology influenced design: the stones of Etruria were not well suited to walling, for example, where earth would suffice, and the need to protect earth walls from rain led to the use of terracotta roofs with wide eaves. Climate, too, likely played an important role, as rising water levels and flooding spurred the construction of *palafitte* and caissons in the Bronze and Iron Age using sophisticated woodworking techniques that would be employed in timber buildings for centuries afterwards (Zamboni 2017; Mistireki and Zamboni 2020; Turfa in press). We should acknowledge that the standard categories of domestic, religious, and civic architecture are not necessarily applicable in early Etruscan history. Funerary architecture existed but likely had political and sacred roles, too. A distinction between private and public architecture cannot easily be recognized at all times either. Again, we need to recognize the gap between the data and the models we apply to its interpretation, the assumptions that bridge it, and how change can be prompted by new data and approaches. Here, we set out the most significant changes and themes in architectural history over the last decade.

### From Huts to Houses

Broadly speaking, Etruscan architecture began with the construction of huts made of wood, wattle, daub, and thatch, with a range of plans, which sheltered all aspects of daily life. In the seventh and sixth centuries BC these structures were gradually replaced by buildings with stone or rubble foundations; walls of mudbricks or pisé, sometimes with timber bracing; and roofs of heavy terracotta tiles, requiring increasingly rectangular plans. This change in building materials and technologies is typically summarized as a shift from huts to houses.

The transition from one dominant building type to another can now be reconstructed as a series of gradual, irregular changes in construction techniques over the course of centuries that allowed for some continuity of traditional behaviors, rather than an abrupt change (Miller 2017). Some methods used to prepare the ground and wall footings, for example, were in use from the Final Bronze Age to the seventh century, while the ongoing use of substances other than stone to infill walls can be seen from at least the eighth to the late sixth centuries. We also know that huts and houses coexisted in settlements for a significant length of time. There is even increasing evidence for the adoption of rectangular foundations and sturdier walls with thatched roofs, challenging the notion that changes to substructures and superstructures occurred simultaneously (Wikander 2017). We still need to explore why houses became larger following changes in materials and design—demographic pressure, a change in the constitution of households, and different sociopolitical uses of residences are all possibilities—and how they were occupied.

The changes wrought by the introduction of tiled roofs have not been underestimated in the past, but our understanding of how such roofs were designed, produced, and installed have advanced notably in the last 11 years. Winter’s comprehensive survey of architectural terracotta decoration in Etruria and central Italy from c. 640–510 BC updated material last synthesized in the mid-20th century and identified common traits that allow elements and roofs to be organized into decorative systems (Winter 2009). The names of these groupings, such as the Veii-Rome-Velletri system, reflect their geographical spread and show the extent to which Etruscan terracottas were connected with those in Latium and Campania through the actions of workshops and patrons. The fact that each system has its own internal grammar furthermore enables the reconstruction of larger designs and even whole roofs from fragmentary evidence.

Winter, following the other methods for terracotta classification developed earlier by Della Seta (phases) and Wikander (typologies), clarifies how changes in the design and manufacture of tiled roofs reveal interaction between central Italic artisans and their counterparts abroad. The independence of Etruscan and central Italic artists and commissioners shines through from the start: although tilemaking technology seems to have been introduced from Greece around the middle of the seventh century BC, it appears likely to have arrived without imagery, meaning that the decoration of the roofs and their use within settlements was driven by indigenous customs. Influences from Corfu, Sicily, Corinth, eastern Greece, and Asia Minor can be discerned in different places at different times but always as part of repertoires that catered to local clients and appeared on a wider range of buildings than in Greece, not least because central Italic artisans made changes that simplified production and reduced costs (Wikander 2017). In time, Etruscan innovations also filtered east to influence roofs in Greece (Winter in press).

In addition to these works, the field has made collective advancements through recent *Deliciae Fictiles* conferences. The fourth conference focused on images of gods, monsters, and heroes, while the fifth examined networks and workshops (Lulof and Rescigno 2010; Lulof et al. 2019). The latter included material from the eastern Mediterranean to further analyze relationships between artisans and production centers in the connected world of which Etruria was part. These meetings remain critical opportunities for contextualizing finds and communicating the strength of the subject as a whole to those working in related areas. Historians have yet to realize the full potential of this material, however, for anchoring social and economic histories (Smith 2019a).

The next step for architectural historians is to reconnect studies of roofs with those of the buildings on which they stood. Our knowledge of roofs has grown so rapidly that arguably it has become slightly separated from that of the rest of the built environment. The famous site of Poggio Civitate, host to an array of Orientalizing and Archaic structures and home to one of the largest examples of residential architecture uncovered to date in Etruria, shows the challenges. Here numerous roofs have been identified that cannot all be assigned to buildings at present. One of the newly discovered Orientalizing buildings, designated EPOC4, may have been the intended recipient of the tiled roof that was being made in the workshop when fire destroyed much of the site in c. 590–580 BC (Winter 2019). The use of tiles of different sizes on the roof of the workshop, perhaps due to an extension or repairs, is a useful reminder that the roofs of buildings could also change without fundamental alterations to plans (Wikander 2017). The process of mentally replacing roofs on lost walls and traces of foundations is clearly worth pursuing; at Satricum, efforts to reconnect the roofs from different phases of the temple on the acropolis with extant foundations have identified a hitherto unknown building (Lulof and Opgenhaffen in press). Given that artisans could not design a roof without knowledge of the size and arrangement of the wooden beams that would carry it, which in turn relied on sufficiently engineered walls and foundations, the design of the building as a whole and its fitness for purpose should be at the forefront of our minds, just as it was for ancient architects.

Housing for the dead must also be woven into these narratives. Although we still lack a handbook or agreed typology for Etruscan tombs, and the diversity of designs may even preclude consolidation, it is undeniable that the quantity and quality of the data show that the living committed considerable resources to housing the dead over a large swath of time. As mentioned earlier, funerary structures became sites of conspicuous consumption and repositories for surplus wealth in the Orientalizing period, when the giant burial mounds known as *tumuli* were introduced to cover sets of multiple burials in chamber tombs in a marked change from earlier burials of one, two, or three individuals in cinerary urns or small inhumations (Becker et al. 2009). The shift to collective burial signals a new conception of social relationships in death and/or life, while its monumental qualities suggest that a new goal, or new method of achieving the same goal, had been widely agreed. The subsequent reduction of investment in funerary architecture during the Archaic period, when temples and other civic projects drew more focus, must likewise indicate new values or concerns (cf. Marzullo 2017).

### Building Communities

Research on civic architecture continues to move beyond discussions of Greek *comparanda* and recognize different Etruscan practices. Roads, ports, fortifications, religious buildings, and town planning can all be examined under this heading as forms of community infrastructure (e.g., Bagnasco Gianni 2018).

Excavations of Iron Age Bologna (Etruscan Felsina) in the last 20 years have yet to be fully published but have the potential to contribute to the range of evidence for communal architecture predating the floruit of cities in the Archaic period. Extensive construction appears to have taken place at Felsina during the eighth century BC, creating water management systems, fortifications, and an enormous wooden complex—more than 120 m long and extending over 6,000 m^2^—that seems to have comprised a series of connected platforms lying outside the limits of the settlement. A working hypothesis is that this structure may have been a civic space for assemblies of a political, military, and religious nature (Ortalli 2013; Santocchini Gerg 2015).

Studies of the building sequences at Marzabotto are slowly overturning the idea that the reorganization of this city in the late sixth/early fifth century into an orthogonal plan replaced a settlement of huts. An intermediate phase in the second half of the sixth century with sacred areas, houses, workshops, and some cemeteries now seems likely, although the layout seems dissimilar from the orthogonal grid thought to derive from the application of Etruscan cosmological and ritual principles (Govi 2014, 2017a). The formalized plan contained plots of different size (between approximately 630 and 900 m^2^) and status depending on their location, each occupied by either an atrium house or a set of separate buildings with a common open-air courtyard. Each unit is also likely to have had an autonomous roof system (Gruška et al. 2017). There is, thus, provision for a more varied urban appearance and subtle hierarchies than the notion of regular streets and tracts of land might first suggest.

Research on the underwater structures at the settlement and sanctuary of Pyrgi are providing more data about Etruscan harbors. The location of wells helps reconstruct the shoreline at Pyrgi in the Etruscan period, lying 70–80 m seaward and approximately 1.5 m below today’s level. By the Archaic period Pyrgi seems to have had two port basins, one in front of where the Santa Severa castle now stands and another closer to the monumental sanctuary, connected to each other by channels (Enei 2008; Michetti 2016). In front of the basin near the castle, two stone platforms formed of boulders on top of sandstone shoals lay on either side of a channel and have been interpreted as Etruscan jetties (Rovere et al. 2010). Such investigations are adding to knowledge of ports derived from work at other sites like Gravisca, Populonia, and Regisvilla.

The construction of monumental temples during the Archaic period has been another fruitful area of study. While buildings were likely sites of cult activity from at least the Iron Age, monumental temples only appeared during the sixth and fifth centuries BC, seemingly replacing residences and tombs as some of the most prominent building projects in settlements and the countryside (Potts 2015). It is unclear whether this change stemmed from an altered view of religion or its role in society, but it is likely to have had significant economic and civic impact. Superlative examples of the new temples include the first and second buildings in the Ara della Regina sanctuary at Tarquinia (Bonghi Jovino and Bagnasco Gianni 2012), which place the city firmly in the vanguard of Etruscan temple design through the use of a massive platform, a high podium, and an elongated central *cella*. These influences from multiple cultures combined to produce a unique Etruscan building. Elements of the plans and particularly the landscaping of the site can be compared to the roughly contemporaneous Temple of Jupiter Optimus Maximus on the Capitoline hill in Rome (Bagnasco Gianni in press), a building that remains the subject of intense study. Different reconstructions of the Archaic Roman temple have been offered (Cifani 2008; Hopkins 2016; Mura Sommella 2009), principally differing on the arrangement of columns and rooms to the rear, but all argue for a temple of colossal size. The theory that the temple may have been smaller and occupied less of the platform has now largely been discarded.

It is clear that the design of Etruscan temples was far from uniform. Although the Roman architect Vitruvius recorded a set of temple proportions that he called the *tuscanicae dispositiones*, they do not exactly match any Etruscan building uncovered to date (Potts 2014–2015). Typologies tend to group approximations together under the heading of Tuscanic or Etrusco-Italic temples, in contrast to those with an elongated *cella* and a full or partial *peripteros* that are generally termed Greek temples, but varieties of both were built and used in Etruria, even side by side at sites like Marzabotto and Pyrgi. In reality temples in Etruria could have *peripteral* and *peripteros sine postico* arrangements, columns that were prostyle or *in antis*, one or three *cellas*, and porticoes or colonnades. We should also be wary of prioritizing plans in our studies when other elements of superstructures could substantially affect a building’s appearance. The Temple of Tinia at Marzabotto, for example, had a peristyle but also a high podium, a frontal staircase, and fewer columns at the front than the back in order to highlight the façade. Other local elements of temple design could include the use of earth and wood, roofs with recessed gables, and statuary placed along the ridgepole (rather than in pediments). The diversity of plans, elevations, and decoration shows that religious architecture was not conservative but highly creative and flexible, reinforcing the idea of religious heterogeneity outlined above.

### Continuity

Continuity and change have long been the poles between which architectural and social histories oscillate. If the architectural history of Etruria has traditionally been characterized by change, however, in terms of periodization and the successive introduction of new technologies and ideologies, recent work has increasingly recognized continuity as well, not just between huts and houses but in the layout and location of certain buildings, in the use of materials, and between cultures that have historically been studied separately.

The relationship between architecture in Etruria, and indeed central Italy, on the one hand and the buildings of Rome on the other has been revisited in the last decade with results that undermine notions of stark division. Arguments for acknowledging the relative autonomy of Roman practices from at least the sixth century BC have been made (Cifani 2008; Hopkins 2016) and countered (Edlund-Berry 2013), but both sides have moved away from envisaging a “decline” in Etruscan architecture in the fifth or fourth century BC followed by the rise of new “Roman” practices. The use of fired earth that passed from tiles to bricks, the employment of arched structures in cisterns and then passageways, select temple designs, a propensity for large-scale landscaping, and the remodeling of roofs and buildings over centuries created a relatively unbroken chain of construction practices through the middle of the first millennium BC. The outcome is that a “Roman” variant of these practices either existed from at least the Archaic period, or that it is now more accurate to speak of a relatively unified field of “central Italic” architecture, without watersheds or cultural rupture, until the last centuries BC.

A specific area in which continuity between the sixth and second centuries BC has been posited recently is domestic architecture. In some ways Poggio Civitate can be viewed as a forerunner of later villas, and in terms of residences on a smaller scale it has been suggested that a canonical house plan emerged in the sixth century and remained in use long enough to shape the development of Republican atrium houses (Jolivet 2011). In addition to the fifth century atrium houses known from Marzabotto, this argument brings further evidence to bear on some continuity of plan, but more examples of Etruscan dwellings from the fourth and third centuries BC would strengthen it further; the sites in Bentz and Reusser (2010) are a start. Scattered finds remain the norm, but houses like the Domus dei Dolia at Vetulonia appear to have been part of Hellenistic Etruscan neighborhoods that warrant further excavation (Grassigli and Rafanelli 2019). For now, the question of how to reconcile Etruscan house plans from the fourth to second centuries BC with mid-Republican examples in Latium and Campania remains open.

The ways in which buildings contributed to social personas and had their own form of identity may be another area of continuity. Roman houses have been studied as a locus for the construction and articulation of identity, and similar ideas have been explored, albeit to a lesser extent, in the Etruscan data. The ideological significance of monumentality has been examined (Thomas and Meyers 2012), as has the concept of buildings as bodies with their own biographies from creation to destruction (Warden 2011, 2012). Continuities between the built and natural environments have also been considered. Similarities between the ritual deposition of bodies and acroterial statues give pause for thought, as does the dismantling or dismemberment of buildings and then the assignment of parts to the gods in votive deposits, akin to sacrifice, and the use of built elements in funerary contexts as symbolic bodies (Warden 2012). Such studies are prompting new consideration of Etruscan buildings as social actors and ways that people mediated their experience of the world. The social role of architecture accordingly comes to the fore once more.

Thus far, we have traced the broad outlines of Etruscan social, political, and religious development, and the settings in which they occurred. We have touched on the economy repeatedly and begin to suggest how some of these ideas might coalesce in a reformulated notion of the Etruscan economy.

## The Etruscan Economy: Institutional or Social?

That the Etruscans collectively were engaged in a substantial and significant array of exchanges internally and externally is evident from any visit to an Etruscan museum or exhibition, as well as all of the studies we have already discussed. From the Late Bronze Age until the Roman period, the Etruscans obtained, produced, and exchanged objects from the mundane to the exotic. Etruscan cities were filled with both the activity and the products of manufacture, and the rural landscape was highly productive (Biella et al. 2017).

Recent work has made significant strides toward developing a greater appreciation of this productive aspect. In the cities, more attention has been given to artisanal activity, as we have noted. In the countryside, field surveys have transformed our understanding of rural settlement patterns over time. We now have a much clearer sense of the contraction after the Late Bronze Age, the steady expansion of settlement thereafter, and then variable patterns of intensification over subsequent centuries.

At some level, it must be the case that the production of an agricultural surplus provided the capacity for Etruscan elites to participate in trade and exchange. As Woolf (2020) has recently pointed out, even in highly urbanized landscapes such as Etruria most people lived outside cities. The ancient economy was fundamentally about agriculture. It is fascinating to see specialized activity in other areas—for instance, iron production in Populonia—partly because it is relatively unusual for a nonagricultural product to dominate in any Mediterranean economy, but one can always find evidence of an agricultural economy as well (Cianferoni 2010).

Yet the mineral wealth of Etruria was certainly a key factor in its economy. Parts of Etruria were rich not only in iron but also copper, silver, lead, antimony, zinc, arsenic, and tin. Populonia was only one of many Etruscan cities exploiting these resources. It is correct to note that “…both the ancient sources and the archaeological data agree in stressing how metallurgy constituted one of the main economic and cultural engines of the Etruscan centers ever since the proto-historical period” (Giardino 2013, p. 733), and we have still fully to appreciate to what extent this placed Etruria into the same kind of economic and ecological world as Athens, with its silver mines, or the gold and silver resources of Macedonia and Thrace (see Williams 2009).

Another basic premise is that the Etruscan economy was an economy of goods. The flow of grain, oil, and wine, supplemented by fruit, vegetables, dairy products, fish, and meat was the core activity, traceable via containers, especially ceramic (Barbieri et al. 2010; Ciacci et al. 2007). Archaeobotanical work is beginning to develop strongly and will in due course, no doubt, intersect with climate change models (Mariotti Lippi and Mori Secci 2007; Mariotti Lippi et al. 2020). Secondary agricultural products included leather, textiles, and implements or decorations made of bone, all of which are attested in numerous excavations. Charcoal was an essential part of metalworking, and there are good reasons for assuming substantial and accelerating deforestation in Etruria (Di Pasquale et al. 2014). Ceramic production was diverse, ranging from coarse ware to very fine *bucchero*, and metals were worked in profusion and with skill to make implements, jewelry, household decorations, and statues (e.g., Ambrosini and Jolivet 2014; Cappuccini 2014; Shipley 2015). The ornamenting of the city and individual houses (apart from roofs) is one of the least well-known elements of the Etruscan archaeological record, but where it can be inferred it was clearly significant (Agnoli et al. 2019; see also Donati 2016).

This means that at any given stage one can see the Etruscan world as object oriented and object rich, as a producer and a consumer. The important question remains though as to what kind of economy this was. The aspects that remain tantalizingly out of reach concern interdependence, scale, institutional organization, and notions of value.

Some interdependence between producers and elite can be assumed, but the precise nature remains unclear. When the landscape refilled with small farms after the settlement of the large plateaus, were the farmers independent or were they bound to elites? If the latter, then did elites increase indebtedness over time? Very little can be said about indebtedness in Etruria, but it is being increasingly studied in Greece, and we have hints of similar processes in Rome (Lerouxel 2015; Zurbach 2017). We suspect that the Etruscans had slaves and freed slaves (*lautni* in Etruscan), with the latter seemingly attested by the Archaic dedication at Campo della Fiera by a freedwoman described above (Capdeville 2002).

The question then becomes one of how interdependence actually worked. If agricultural surplus in some ways flowed to an elite, how was it converted into the capacity to procure luxuries from across the Mediterranean? Etruscan coinage began relatively early in the fifth century BC, but it was rare and sporadic; there is no evidence for a monetized economy. One might imagine that agricultural surplus was used to secure labor and military service, although that may be a rather grand term for raiding and piracy. Additionally, marriage dowries and the like may have been opportunities for elite exchange and display. Some highly similar sets of material from Orientalizing “princely” graves in different locations, for example, the Tomba Regoloni Galassi in Caere and the Tomba Bernardini in Praeneste, might reflect the division of a set of material in connection with a wedding.

It is also possible to suggest that we see a gradual spread of wealth to support local elites in the countryside. This might explain local tomb groups and increasingly complex land ownership arrangements, which we can glimpse later in the *Tabula Cortonensis* from the second century BC (Agostiniani and Nicosia 2000; Bruschetti et al. 2015, p. 46). However, we desperately need more and better excavations of rural sites. As an indication of what can be done, a detailed investigation of a rural site at Marzuolo in the Roman period makes the case that innovation was driven by local smallholders, instead of elite landowners, who were limited by a lack of capital for investment; this restriction opened the way for predatory landowners to take over facilities and curtail further innovation (van Oyen 2020). This case study may point to a wider pattern, stretching back in time, of persistent attempts to raise local capacity that were limited by resources and then subsumed into relatively conservative economic strategies. Intriguing questions would then be whether this was replicated at local and community levels or not, and if interdependence meant that some communities found it difficult to rise to a more significant level of economic prosperity. Strategies for escaping these constraints could have included developing natural or supernatural resources, for instance shrines and sanctuaries.

Such a scenario raises a significant problem, however, about the scale of the economy. At what stage, if ever, did the Etruscans create genuine significant growth and scale in production? This question has been posed for the economy of imperial Rome but seldom for Etruria. Even Poggio Civitate, for all its size and wealth in the Archaic period, may not have had a strong export production, although it may have had some mineral wealth (Giardino 2013, p. 731). Riva (2017) has suggested that even the export of wine to southern France in the sixth century BC that is often seen as a sign of significant surplus production may equally be characterized as prestige exchange, as often we struggle with estimating scale of production given the problems of survival of archaeological material. Since the fifth century was a period of relative contraction—at least in the south and along parts of the coast—and the third century brought Roman conquest, it becomes difficult to identify a significant period of economic growth. In the absence of helpful literary evidence, it is equally difficult to identify institutions that helped overcome or ameliorate inevitable frictions in trade.

The apparent absence of an institutional framework for Etruscan trade may, of course, be another consequence of our evidence. This should not stop us asking questions and exploring potential answers. How was metallurgy in Populonia organized? What role did temples play, for example, in agricultural production and distribution? Should we envisage a series of weak or fragile states with powerful clans within? The idea that extended lineage groups drove economic performance has become increasingly attractive, and it fits with the one constant in Etruscan archaeology that is the persistence—with gaps and hiatuses—of significant familial continuity and auto-celebration. On the other hand, four different objections could be raised to the idea that the Etruscan economy was based on the performance of extended lineage groups. First, this theory may overstate the economic performance of any small city-state (in other words, scale was always difficult to achieve). Second, it may understate the role of artisans and individuals (in other words, the search for institutions misses the reality of multiple levels of connectivity). Third, it may understate constraints and initiatives at the level of the community, both as an arena in which elite performance was expected and demanded and also as a generator of large-scale projects (building projects are an obvious but not unique example). Fourth, it assumes a continuity of value assumptions over time (that is, that the economic values of later periods are constants to be found in earlier times).

The critical question for understanding ancient economies remains the question of the location and nature of the value at stake. Any assumption that the Etruscans valued the maximization of profit may be far from the reality. This was an economy performed and created in a world transfused with immanent gods and where behaviors were scrutinized and calibrated against shifting local, regional, and international standards. The contraction of exceptional funerary display in the later sixth and fifth centuries may reflect wider tendencies toward frugality and a different notion of appropriate behavior (see above). We see this particularly clearly in Rome and also Sparta (Colonna 1977; van Wees 2017). As far as we can tell, this relaxed again in the fourth century, and we can ask if there might have been any structural incentives for economic growth or ideological limits.

The general view tends to be that Etruscan city-states were governed in a relatively oligarchic fashion (again, this is perhaps the product of skewed evidence). An alternative might be that extensive slave labor permitted significant citizen leisure, and it then becomes an interesting question as to how far down the social scale this may have extended. In any combination of these scenarios, the question remains what transformation of value may have occurred to encourage accelerations in production either through elite extraction to sustain elite behavior, or through a broader social desire for increased tangible wealth and luxury.

Here the critical evidence relates to stories of slave wars or uprisings, specifically at Arretium and Volsinii, opposed in both instances by aristocrats with support brought in from outside, in the first case Tarquinia, and in the second Rome (Benelli 2013). The challenge is to know how to read this evidence. Were these actually slaves, or is this a pejorative description of a nonelite class? What does victory look like? In the case of Volsinii, the old city was destroyed and a new one created under Roman influence, but names in the new foundation seem to have significant continuities with the earlier one. It is still unclear whether entrenched aristocracies were the norm or the exception and how far their power extended.

Ultimately, much about the Etruscan economy will remain obscure simply because we lack sufficient evidence. The challenge is then twofold: to test many different kinds of models and approaches, and to look for more archaeological sites that could disprove or confirm them. We need to know more about rural Etruria and its small farms, more about industries in Etruria, and more about Etruscan ports (e.g., Bagnasco Gianni and Fiorini 2018). We need to think harder about the consequences of the movement of objects. It is often noted that ceramics and other goods were mobile, but the significant advances in understanding mobility more generally have not perhaps been sufficiently applied (see Isayev 2017). There may also be value in long-term analysis and comparisons, for instance, between the preconquest period and our increasing knowledge of late Roman Etruria, when the structures enabled and empowered by the reach of Roman imperialism were either not in place or beginning to falter. Comparisons with Rome then return us to the start and the question of how Etruscology now relates to other classical cultures in the minds of the academy and the public.

## Display and Dissemination: Reaching New Audiences?

In the last decade, researchers have spent much time and effort attempting to raise the profile of the Etruscans. The phenomenon of the blockbuster Etruscan exhibition has accelerated, with shows in Paris, Leiden, Amsterdam, Karlsruhe, Seoul, Rome, Naples, Chicago, and Luxembourg among others; at the same time numerous handbooks and introductions to the Etruscans have appeared in English. Both endeavors have given the Etruscans renewed prominence outside their homeland, but scholars should now confront questions about the ethics, sustainability, and audience of their work.

Displays of the brightly painted tombs, glittering ornaments, and striking sculptures of Etruria, as well as the persistent if now unfounded trope of the “mysterious” Etruscans, have drawn crowds for centuries. Temporary exhibitions offer useful opportunities for museums to display items usually housed in storerooms, often in conjunction with loans from Italian institutions. Appeal also lies in the fact that in comparison with Greece and Rome—and their historical inheritance at times invoked to different nationalist, colonial, and patriarchal ends—the Etruscans seem to be a *tabula rasa* able to carry marketable storylines. They were a culture of eminent women (Leiden and Amsterdam), of multicultural and polyethnic communities (Karlsruhe and Chicago), of joyous lifestyles (Paris), of protometropoleis (Lens), of intriguing belief systems (Luxembourg), of material sophisticates and the heralds of Roman glory (Seoul). Coupled with lavish artifacts, this flexibility has made them a regular feature of international exhibition calendars. Over the same period, high-profile Etruscan galleries have been redesigned, for example, at the Getty Villa and the Louvre, following reinstallations of the Etruscan displays at the Metropolitan Museum of Art and the Penn Museum. Such projects play a critical role in revitalizing museum holdings and drawing visitors’ attention.

Yet despite this investment in exhibiting the Etruscans, the systems that lie behind the formation of the collections have been obscured or ignored too often. A close symbiosis between archaeology, collecting, and museums has characterized Etruscan studies since the 19th century and explains not only the presentation of the Etruscans in Italian museums but also the diasporic spread of goods from Etruscan sites across the world. But the darker side of this relocation of material—namely the desire for artifacts that drives looting, the market for illicit antiquities, and forgeries—has received far less attention than the display of the end result. Museum labels and catalog entries give various weight to discussions of style and use but tend to skirt unknown provenance or problematic acquisition histories, with notable exceptions (e.g., Agnoli et al. 2019; Gaultier et al. 2018). We would argue that more research into, and honesty about, the origins of museum collections could make Etruscan material a model for those working in museum studies, art history, and archaeological heritage.

The legacy of temporary shows also would now repay consideration. The outcome has not always been a higher profile for Etruscan material in galleries, university curricula, or the cultural landscape beyond Italy. Whereas permanent exhibits serve as collective memories, temporary exhibitions should be catalysts for the production of new knowledge, generated by the development process and disseminated through staging and related publications. But despite the regular issuing of catalogs and related edited volumes, including the useful apparatus published in conjunction with exhibitions, such as *Maestri di scrittura* (Bruschetti et al. 2015), *Die *Etrusker (2017), and *Etruschi* (Bentini et al. 2019), few if any recent Etruscan shows have achieved the scholarly impact of exhibitions like *La grande Roma dei Tarquini* (Cristofani 1990). Somehow progress from concept to display to new perspectives has been disrupted.

Contemporary events could see this chain of knowledge reassessed. As museums the world over are forced to reckon with the rising costs of international loans and insurance, the environmental cost of moving objects round the globe and relying on visitors to travel to artistic centers, in tandem with falling visitor numbers, issues with endowments and sponsorship, and, finally, the uncertainty created by the closures and social distancing requirements caused by Covid-19, some museum commentators are asking if the age of the blockbuster exhibition is over. Etruscan material will not be exempt from such reevaluations and will likely have to fight for its share of resources (cf. Di Gennaro 2009). Philanthropy will become even more important, and significance beyond closing day will need to be maximized.

One way forward may be to stage exhibitions that are driven by research rather than esthetic experience. Instead of seeking loans, museums could use their collections to show how artifacts can drive and answer targeted questions about the past and generate alternative histories (cf. Bagnasco Gianni 2008). Comparison with other cultures could bring new perspectives and context. Controversy and complexity should be acknowledged. Museum interpretation would become more important in displays, but it is a renewable resource. This suggestion does not preclude spectacular or touring exhibitions but considers how opportunities and benefits could be spread more evenly. This review has sought to offer a number of provocations, and there are many more that could usefully generate a more focused symbiosis between research and display.

Over the same period that researchers have worked on exhibitions and gallery renewals, they have also contributed to multiple handbooks, companions, and introductions to the Etruscans in English (Bell and Carpino 2016; Lulof and van Kampen 2011; Naso 2017; Riva 2020; Shipley 2017; Smith 2014; Turfa 2013), as well as an important one in Italian (Bartoloni 2012). Together with works issued earlier this century (e.g., Camporeale 2004a; Haynes 2000; Izzet 2007), the market is surely replete for now. Each is aimed at different readerships, but inevitable overlap along with wide variation in length and price has forced libraries and instructors to make difficult decisions. Together they have succeeded in making a wealth of new material accessible to Anglophone audiences and significantly increased the ease with which students outside Italy can start to study the Etruscans. In-depth study, in contrast, still requires engagement with publications in Italian and a relative lack of specialist monographs: edited books and conference proceedings remain the primary vehicles for disseminating Etruscan scholarship, and readers typically have to follow repetitions and updates of specialist material over decades rather than consult extended treatments or syntheses. The disjunction between Anglophone and Italian publication strategies is more apparent in Etruscan than Roman archaeology and, thus, cannot be attributed solely to the culture of their homeland. We consequently finish by reflecting on the related issues of intended audience and the future of the field.

The outpouring of activity that we have just surveyed suggests, on the one hand, a healthy and far-reaching interest in the Etruscans. On the other, one could ask if it has enticed any new adherents among those already working on the classical world or members of the public interested in the past, or has it changed perceptions of the field more broadly. Has respect (or its measurable corollary, funding) increased with accessibility? Is Etruscology successfully making a case for its continued existence in a world where the allocation of scholarly resources is being ever more contested?

If the answer to these questions is no, then perhaps it is a long game with results yet to come. Alternatively, Etruscologists could benefit from critical reflection on how research is formulated, who it is intended to reach, and how it can advocate for its relevance. Proponents could become more strident in signaling what their subject can bring to the study of the ancient world, to humanities and the social sciences. Trying to attract new audiences to Etruscan conferences, courses, and shows may be less effective over time than taking the subject to them. Courses on Roman art would be stronger for including Etruscan antecedents, especially on topics such as wall painting, sarcophagi, and portraiture. Roman architecture can be evaluated properly only in comparison with practices in central Italy before the middle republic. Analyses of ancient religion would benefit from consideration of Etruscan materiality, and models of urbanism and ancient economies need to account for Etruscan experience to claim any widespread validity. The potential for Etruscan archaeology to grow as an international and interdisciplinary subject is there with reorientation.

## Conclusion

Throughout this survey, we have sought to highlight themes that bring the Etruscans into dialog with other cultures, both in classical antiquity and across the globe, and to show that there is potential for more integration through targeted research; our aim has been to look not just backward but forward. As it stands, the discipline currently has the choice of accepting relegation within classical studies due to its lack of extant texts—note, not because the Etruscans were an illiterate society, but one with a vibrant literary culture that has not survived through accidental and deliberate loss—or working harder to signal the rich material that exists and the opportunities it offers scholars brave enough to engage with it. Dismantling insularity, engaging with comparative debates, and being transparent about history may be challenging, but it could revitalize research and bring new life to the field. Future generations could then enjoy a subject that has not just survived but thrived.
